# Serum Uric Acid and Bone Health in Middle-Aged and Elderly Hypertensive Patients: A Potential U-Shaped Association and Implications for Future Fracture Risk

**DOI:** 10.3390/metabo15010015

**Published:** 2025-01-03

**Authors:** Shuaiwei Song, Xintian Cai, Junli Hu, Qing Zhu, Di Shen, Huimin Ma, Yingying Zhang, Rui Ma, Pan Zhou, Wenbo Yang, Jing Hong, Nanfang Li

**Affiliations:** 1 Hypertension Center, People’s Hospital of Xinjiang Uygur Autonomous Region, Urumqi 830001, China; 2 Xinjiang Hypertension Institute, Urumqi 830001, China; 3 NHC Key Laboratory of Hypertension Clinical Research, Urumqi 830001, China; 4 Key Laboratory of Xinjiang Uygur Autonomous Region “Hypertension Research Laboratory”, Urumqi 830001, China; 5 Xinjiang Clinical Medical Research Center for Hypertension (Cardio-Cerebrovascular) Diseases, Urumqi 830001, China

**Keywords:** serum uric acid, hypertension, bone mineral density, FRAX score, osteoporosis

## Abstract

Background: The influence of serum uric acid (SUA) on bone metabolism, as suggested by previous studies, remains a contentious issue. SUA plays a complex role in bone health and hypertension, making it challenging to discern its impact on the skeletal status of middle-aged and elderly hypertensive patients. This study aims to elucidate the effects of SUA on bone health, with a particular focus on its association with osteoporosis and the risk of fractures. Methods: Multiple linear regression analyzed SUA levels against bone mineral density (BMD) and future fracture risk. Additionally, multivariate logistic regression was used to examine the association between SUA and osteoporosis. Dose–response relationship analysis was conducted using generalized smooth curve fitting (GSCF) and restricted cubic spline (RCS) methods. Results: With the exception of the total femur region, SUA and BMD showed a positive connection. GSCF analysis revealed an inverted U-shaped relationship between SUA and BMD, alongside a U-shaped trend with FRAX scores. Moreover, RCS analysis indicated a U-shaped relationship between osteoporosis risk and SUA levels, with higher risks identified in the first and third tertiles compared to the second tertile. Conclusions: In individuals with middle-aged and older hypertension, SUA is substantially linked to bone health. The identification of an inverted U-shaped relationship with BMD and U-shaped relationships with FRAX scores and osteoporosis risk highlights the nuanced influence of SUA. These findings suggest that both low and high SUA levels may adversely affect bone health, emphasizing the need for further research.

## 1. Introduction

Osteoporosis, characterized by diminished bone mass and bone mineral density (BMD) along with microstructural deterioration, is a chronic metabolic bone disorder that enhances skeletal fragility and the propensity for fractures [1]. Recent studies have reported a significant rise in osteoporosis among hypertensive patients, a trend that has raised substantial concern [2,3,4]. As the global prevalence of both hypertension and osteoporosis continues to climb, identifying the risk factors for osteoporosis within the hypertensive population has become a critical public health issue [5,6].

Traditionally, the determinants of bone loss and osteoporosis have been linked to lifestyle factors such as smoking, inadequate nutrition, insufficient calcium intake, and a sedentary lifestyle, as well as the use of specific medications like steroids and personal behaviors such as alcohol consumption [7,8]. Additionally, non-modifiable factors, including advancing age, female gender, and a positive family history of osteoporosis, play a significant role [5]. However, a growing body of research has started to support the hypothesis that oxidative stress may be an underlying mechanism for age-related bone loss, possibly by enhancing osteoclast-mediated bone resorption [9,10,11].

Uric acid (UA), a purine metabolite once thought to be merely an excretory byproduct, has recently garnered attention for its role in health and disease. Serum uric acid (SUA), the primary form of uric acid in the bloodstream, is now known to be closely linked to the development of various diseases. Elevated SUA, in particular, has been strongly associated with the pathogenesis of gouty arthritis and kidney stones [12,13,14]. On the other hand, SUA is also recognized for its antioxidant properties and is believed to exert protective effects in conditions such as cancer and several neurological disorders [15,16,17]. The role of SUA in bone metabolism has also gained attention, with evidence suggesting that it may play a protective role in bone health under normal conditions [18,19,20]. Its antioxidant capacity is thought to protect bones under normal physiological conditions [19]. However, disruptions in SUA metabolism can trigger an inflammatory cascade and oxidative stress, which may impair osteoblast-mediated bone formation while promoting osteoclast activity, ultimately contributing to osteoporosis [18,19,20].

Given the impact of UA on bone health and the fact that SUA serves as the primary manifestation of UA metabolism, its measurement is both concise and readily accessible. Therefore, it is crucial to explore the effect of SUA on bone health, particularly in middle-aged and elderly hypertensive patients. Several factors can influence SUA levels in this population, including hypertension itself, medications, and concomitant metabolic disorders. Moreover, since this group is at high risk for osteoporosis, understanding the complex relationship between SUA and bone health in hypertensive patients is of clinical significance.

This study seeks to explore the relationship between SUA and bone health in middle-aged and elderly hypertensive patients, focusing on its implications for osteoporosis and fracture risk. These insights are expected to enhance the risk assessment of bone health in hypertensive patients and inform the development of targeted preventive and therapeutic strategies. Moreover, a deeper understanding of SUA’s role in bone metabolism may reveal new therapeutic avenues in the management of osteoporosis.

## 2. Materials and Methods

### 2.1. Study Population

**Figure 1:**
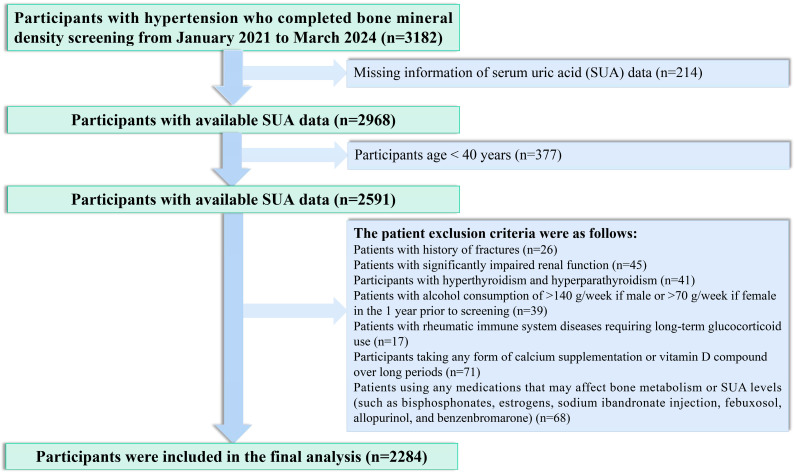
The flow chart of participant selection.

From January 2021 to March 2024, we enrolled hypertensive patients at the Xinjiang Hypertension Center who had completed BMD testing. Out of these, 3182 initially met the criteria. To study the impact of SUA on bone health, we first excluded individuals without complete SUA data. We also excluded those under 40 years old, as osteoporosis mainly affects middle-aged and elderly individuals. Furthermore, participants with a history of fractures, severe kidney dysfunction, hyperthyroidism, hyperparathyroidism, or chronic excessive alcohol consumption were excluded to control for factors influencing bone health. Finally, patients on medications affecting bone metabolism or SUA levels, including glucocorticoids, calcium, and vitamin D supplements, were also excluded. After these criteria, 2284 participants remained in the study (Figure 1).

Ethical approval (KY2022080905) was granted by the People’s Hospital of Xinjiang Ethics Committee, with all participants providing informed consent.

### 2.2. Collection and Definitions of Variable Information

Demographic, lifestyle, and medical data were extracted from the electronic medical records of participants. Anthropometric measurements, including body mass index (BMI), systolic blood pressure (SBP), and diastolic blood pressure (DBP), were measured by a professional nurse using standardized methods. Definitions for current smoking and alcohol consumption, along with their specific daily amounts, are detailed in the Supplementary Material. Participants fasted overnight and had their venous blood collected the following morning. Laboratory indicators, including serum ions, parathyroid hormone (PTH), 25-hydroxyvitamin D, liver enzymes, lipid profiles, creatinine (Cr), alkaline phosphatase (ALP), thyroid stimulating hormone (TSH), fasting plasma glucose (FPG), and SUA, were measured using an automated analyzer, following clinical guidelines and hospital precedents [21,22,23]. Estimation of glomerular filtration rate (eGFR) was calculated using the CKD-EPI formula. The participants’ medical histories, including hypertension, primary aldosteronism (PA), diabetes mellitus (DM), coronary heart disease (CHD), and cancers, are described with diagnostic criteria provided in the Supplementary Material.

### 2.3. BMD Assessment

BMD was measured by dual-energy X-ray absorptiometry (DXA) in the lumbar and femoral regions, with scans performed by a radiologist using a bone densitometer. Measurement protocols are detailed in the Supplementary Materials.

### 2.4. FRAX Scores

The FRAX score, developed by the World Health Organization, is a tool designed to predict a population’s ten-year fracture risk based on multiple risk factors. In this study, we utilized the China-specific FRAX assessment tool to estimate the probability of major osteoporotic fractures (MOF) and hip fractures (HF) over the next ten years [24]. Additional FRAX score details are available at www.shef.ac.uk/FRAX (accessed on 25 April 2024).

### 2.5. Study Outcomes

The T-score at each site was calculated using the DXA. Osteoporosis was defined as a BMD T-score of −2.5 standard deviations or lower in any region of the femur or lumbar spine [25,26,27]. The probability of participants developing MOF and HF over the next ten years was estimated using the China-specific FRAX score [24,28,29].

### 2.6. Statistical Analysis

Participants were stratified into tertiles based on SUA levels. Multicollinearity was confirmed absent with variance inflation factors (VIFs) below 10 (Table S1). Multiple linear regression assessed the correlation between SUA levels and BMD and the risk of future fractures. Moreover, the calculation of odds ratios (ORs) for osteoporosis was executed using multiple logistic regression analysis. Dose–response relationship analysis was performed using generalized smooth curve fitting (GSCF) and restricted cubic spline (RCS). Gender stratification was integrated into the RCS to analyze the influence of turning on osteoporosis development in both males and females. Conclusively, a battery of rigorous subgroup analyses and sensitivity tests were meticulously conducted to substantiate the robustness of the observed findings. Statistical analyses were conducted in R version 4.2.2, with significance set at *p* < 0.05.

## 3. Results

### 3.1. Baseline Characteristics

Table 1 outlines the baseline characteristics of 2284 participants, categorized by tertiles of SUA. Contrary to the T1 group, participants with higher SUA levels were predominantly younger and male. These groups also exhibited significantly higher BMI and DBP. Additionally, they were more frequently current smokers and drinkers. Among female participants, the incidence of menopause was notably lower in these groups. Laboratory tests highlighted significant differences across groups in several biomarkers, including serum potassium, PTH, serum phosphorus, ALT, AST, TC, TG, Cr, ALP, TSH, FPG, and SUA. Participants with elevated SUA levels also exhibited a lower prevalence of PA, DM, and cancer and were more frequently prescribed diuretics and angiotensin-converting enzyme inhibitors or angiotensin receptor blockers (ACEIs/ARBs), with a lower likelihood of using oral hypoglycemic agents. Significantly, there were marked differences in BMD and the prevalence of osteoporosis among the groups. We further grouped participants based on osteoporosis status, finding that the differences between groups were largely consistent (Table S2).

### 3.2. Effect of SUA on BMD

**Figure 2:**
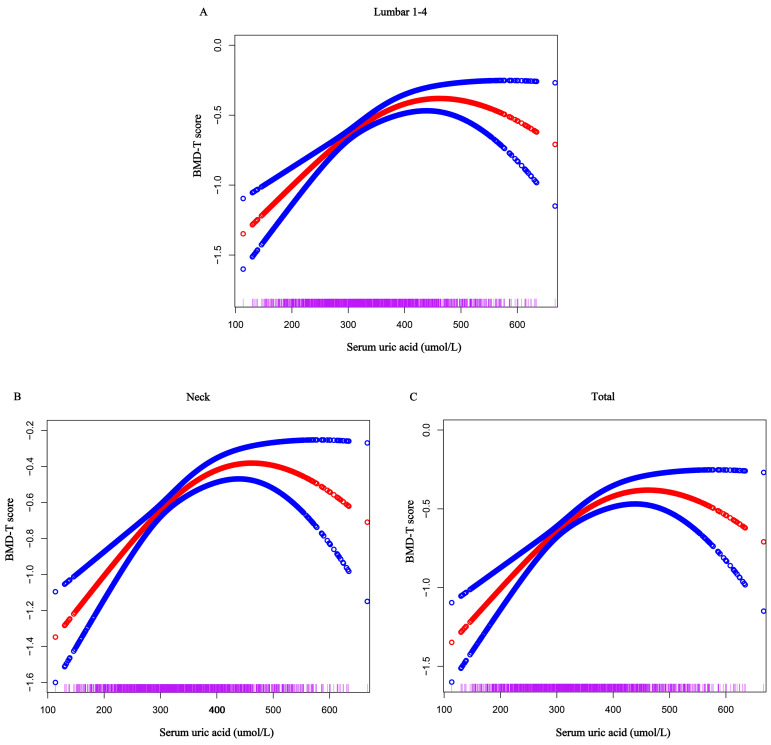
Association between serum uric acid and bone mineral density. Solid red line represents the smooth curve fit between variables. Blue bands represent the 95% confidence interval from the fit. (**A**) Lumbar 1-4, (**B**) Neck, (**C**) Total.

Linear regression showed a positive link between SUA levels and BMD in the lumbar spine and femur. This association was consistent across SUA tertiles, with higher T-scores for BMD in T2 and T3 compared to T1. However, in the fully adjusted model 4, while the positive correlation persisted across most sites, no significant correlation was found at the total femur (Table 2). Furthermore, GSCF analysis revealed an inverted U-shaped relationship between SUA levels and BMD at each site (Figure 2). Threshold analysis of these curves identified specific SUA levels beyond which the protective effects on BMD were lost. For instance, at lumbar 1-4 and the femoral neck, the protective effects diminished when SUA levels exceeded 324 µmol/L (β, 0.009; 95% confidence interval [CI], −0.004, 0.022) and 366 µmol/L (β, −0.005; 95% CI, −0.015, 0.006), respectively. Notably, at the total femur site, a negative correlation with BMD emerged when SUA levels surpassed 365 µmol/L (β, −0.011; 95% CI, −0.022, −0.001), suggesting a potentially detrimental impact on femoral health at higher SUA levels (Table S3).

### 3.3. Relationship Between SUA Levels and FRAX Score

**Figure 3:**
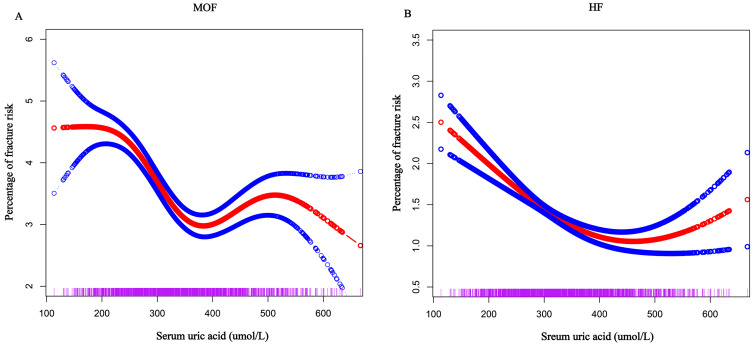
Association between serum uric acid and FRAX score. Solid red line represents the smooth curve fit between variables. Blue bands represent the 95% confidence interval from the fit. (**A**) MOF, (**B**) HF.

Similarly, our study revealed a significant impact of SUA levels on future fracture risk, particularly in relation to the FRAX score. Multiple linear regression analyses demonstrated a negative association between SUA and both MOF and HF, regardless of whether SUA was considered a continuous or categorical variable (Table 3). Additionally, GSCF results indicated a clear U-shaped relationship between SUA levels and the risk of these fractures (Figure 3). Threshold analysis further showed that when SUA levels exceeded 365 µmol/L, the protective effect of SUA against MOF was lost. Notably, at a SUA level of 370 µmol/L, a positive correlation with HF emerged, suggesting a potential adverse impact on bone health and an increased risk of future fractures (Table S4).

### 3.4. Relationship Between SUA Levels and Osteoporosis

**Figure 4:**
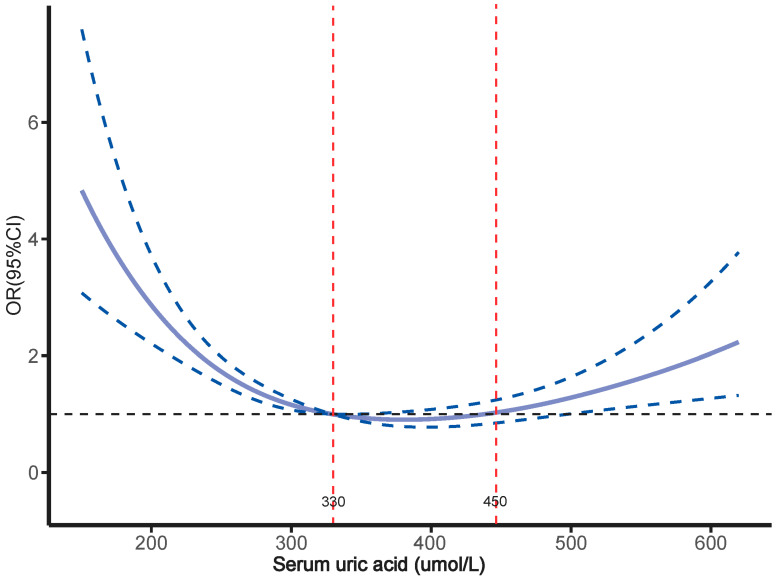
Dose–response association between serum uric acid and risk of osteoporosis.

**Figure 5:**
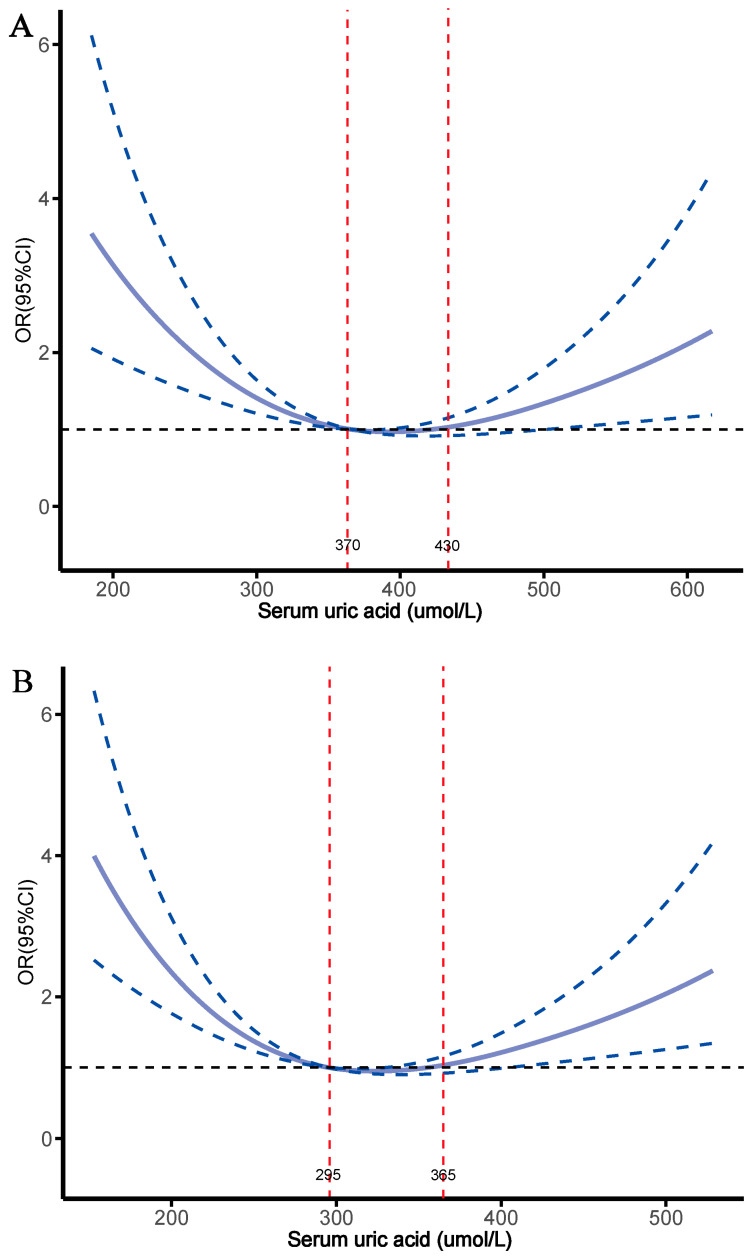
Dose–response association between serum uric acid and risk of osteoporosis under gender stratification. (**A**) male, (**B**) female.

In analyzing the impact of SUA on osteoporosis, multifactor logistic regression revealed a significantly higher risk of osteoporosis in the T1 and T3 groups compared to the T2 group, with ORs of 3.057 (95% CI, 2.386–3.917) and 1.602 (95% CI, 1.232–2.082), respectively. After full adjustment, this elevated risk remained, with the T1 and T3 groups showing 2.427 times and 2.146 times greater risk of osteoporosis compared to the T2 group, respectively (Table 4). The dose–response relationship between SUA levels and osteoporosis, assessed through RCS analysis, revealed a significant U-shaped association. Specifically, when SUA levels were below 330 µmol/L and above 450 µmol/L, there was a notable increase in the risk of osteoporosis (Figure 4). Additionally, a two-stage analysis using the RCS critical turning point was conducted. Below 330 µmol/L, each 10 µmol/L increase in SUA was associated with a 9.7% decrease in osteoporosis risk. However, at or above 330 µmol/L, each 10 µmol/L increase in SUA was linked to a 3.5% increase in osteoporosis risk (Table S5). Importantly, gender stratification revealed that the U-shaped relationship persisted in both men and women (Figure 5). Threshold analysis showed a significant increase in osteoporosis risk when SUA levels were below 295 µmol/L and above 365 µmol/L in females and below 370 µmol/L and above 430 µmol/L in males (Figure 5 and Tables S6 and S7). These findings further highlight the U-shaped relationship between SUA levels and osteoporosis in middle-aged and elderly patients with hypertension, with both too-low and too-high SUA levels likely to increase the risk of osteoporosis.

### 3.5. Subgroup and Sensitivity Analysis

To validate our findings, we undertook rigorous subgroup and sensitivity analyses. Initially, we performed subgroup analyses based on various factors, including participants’ sex, age, BMI, smoking status, and menopausal status (Tables S8 and S9). These analyses aimed to explore further the impact of SUA on bone health across different subgroups. The results revealed trends consistent with the overall findings, reinforcing the conclusion that the effects of SUA on bone health are not influenced by various stratification factors. Furthermore, we conducted extensive sensitivity analyses to ensure the robustness of our findings. First, recognizing the potential influence of malignancy on bone health, we excluded all participants with a history of cancer. Despite this exclusion, our results remained consistent (Table S10). Next, given the adverse effects of 25-hydroxyvitamin D deficiency on bone health, we excluded relevant patients, and the findings were still consistent (Table S11). Additionally, considering the potential bone-related advantages of obesity, we removed participants with obesity from the analysis, which led to largely unchanged results (Table S12). Lastly, understanding that some antihypertensive drugs might negatively affect SUA metabolism, we excluded participants who used diuretics, and this exclusion did not significantly alter the results (Table S13). Further separate analyses of participants taking serum uric acid-lowering medications yielded results that also remained consistent (Table S14). In conclusion, these findings consistently support an association between SUA and bone health issues in hypertensive patients, independent of other factors. This strengthens our conclusion that lowering serum uric acid and maintaining it within a certain range may significantly contribute to preventing osteoporosis and fractures.

## 4. Discussion

The significance of SUA in bone metabolism, particularly among middle-aged and elderly hypertensive individuals, is profound. Our study is the first to thoroughly examine the impact of SUA on bone health within this specific demographic. Given the potential for SUA levels to vary over time, we have chosen to analyze the most current SUA levels of our study participants. This approach provides a more accurate representation of the patient’s current health status and their immediate risk for fractures. We identified a robust U-shaped correlation between SUA levels and the risk of osteoporosis, a relationship that persisted even after rigorous adjustment for numerous confounding factors. Our findings suggest that within an optimal range, SUA concentrations may confer a protective effect on BMD, thereby reducing the risk of osteoporosis and future fractures. However, this protective effect has its limits. When SUA levels exceed a critical threshold, the benefits diminish, and both very low and very high SUA levels negate this protective influence. These insights highlight the therapeutic potential of maintaining SUA within a target range, as reflected by the current levels, which is crucial for the management of bone health in hypertensive patients.

SUA, a crucial product of human metabolism, has been extensively studied due to its abnormal concentration changes being associated with numerous diseases. Elevated SUA levels, known as hyperuricemia, are a key component of metabolic syndrome [30,31]. This syndrome is intricately linked with various metabolic disturbances, including obesity, hypertension, hyperlipidemia, and hyperglycemia [32,33,34]. Traditionally, monitoring SUA levels has primarily focused on assessing the risk of gout and cardiovascular diseases [12,35]. However, recent studies have begun to reveal that the role of SUA extends well beyond these traditional associations, highlighting its broader implications.

Although the physiological function of SUA in the body is not yet fully understood, emerging studies suggest its potential influence on bone health [36,37,38,39,40]. For example, research involving Chinese adolescents has identified an inverted U-shaped relationship between SUA levels and BMD, indicating that moderate SUA levels may benefit bone health, while excessively high levels could be harmful [37]. This finding is consistent with studies from Mexico, where high serum SUA levels have been linked to an increased risk of bone loss, underscoring the complex role of SUA in maintaining skeletal health [36]. Additionally, research led by Ma et al. has shown that SUA levels within the normal range may provide a protective effect against osteoporosis, reinforcing the idea that elevated SUA levels are associated with a higher risk of osteoporosis [39]. Meanwhile, in the relationship between SUA and fracture, it has also been found that urico-lowering therapy to lower serum urate to target guideline-based levels is strongly associated with a reduced risk of fracture in patients with gout [41,42]. In contrast, a study from Taiwan offers a different perspective, reporting no significant correlation between SUA levels and BMD in middle-aged men, nor any association with bone protection [38]. These discrepancies may arise from variations in population demographics and study sizes, highlighting the need for further research to clarify the effects of SUA on bone health. Unlike previous studies, our research focuses on middle-aged and elderly hypertensive individuals, a demographic often affected by both hypertension and osteoporosis. Utilizing a large sample size and comprehensive clinical data, our investigation is the first to examine the impact of SUA on bone health within this specific group. Our key finding—that the risk of osteoporosis significantly increases when SUA levels fall below 295 µmol/L or rise above 365 µmol/L in females and fall below 370 µmol/L or rise above 430 µmol/L in males—is crucial for informing future preventative strategies for osteoporosis in hypertensive patients. Maintaining SUA levels within the optimal range may be beneficial in reducing the incidence of osteoporosis. These findings emphasize the need to lower SUA levels in participants with high SUA levels while appropriately raising SUA levels in those with low levels. This can be achieved not only through the use of relevant medications but also by consuming foods rich in purines, which can significantly improve SUA metabolism and increase SUA levels [43,44,45]. Accordingly, this study consistently underscores that maintaining SUA levels within the optimal range may help reduce the incidence of osteoporosis and fractures.

The influence of SUA on bone health is a multifaceted and intricate biological process governed by a complex interplay of mechanisms. Under physiological conditions, SUA functions as an antioxidant, neutralizing free radicals in plasma and thereby providing a protective effect on bone health [19,46]. However, the relationship between SUA levels and bone health is nuanced, with both exceedingly low and high levels exerting adverse effects, as demonstrated by the distinct U-shaped association with osteoporosis observed in this study [18,47,48,49,50,51,52]. SUA’s antioxidant role is instrumental in mitigating oxidative stress, which, when unchecked due to low SUA levels, can escalate, leading to bone cell damage [49,51,53]. Moreover, SUA actively participates in bone metabolism, including the stimulation of osteoblast activity—key cells responsible for bone formation. Suboptimal SUA levels may, therefore, impede osteoblast function and contribute to a reduction in BMD [50]. Additionally, SUA may affect the absorption and metabolism of essential minerals such as calcium and phosphorus, whose normal levels are essential for bone health [50,54]. Furthermore, SUA is involved in maintaining bone quality, and insufficient levels can impair bone tissue structure and function, thereby increasing the risk of osteoporosis [51,55]. Conversely, persistently high SUA levels can also negatively affect bone health. Elevated SUA can trigger an inflammatory response, increasing osteoclast activity, which in turn raises the risk of osteoporosis and fractures [20,27,29,37]. It can also interfere with the normal function of bone cells, inhibiting their formation and proliferation, resulting in bone loss and osteoporosis [48,56,57]. Additionally, high SUA levels can disrupt intracellular calcium ion balance, impairing the mineralization process and further compromising bone stability [19,52,58]. Furthermore, elevated SUA can affect the endocrine system, particularly estrogen levels, thereby exacerbating the risk of osteoporosis [59,60,61,62,63]. Finally, UA is the end product of purine metabolism. Purines, purine nucleotides, and nucleotides not only affect blood pressure but also skeletal physiology through their receptors, which may lead to pathologic changes in the skeleton, increasing the risk of skeletal deterioration [64,65,66]. Taken together, these mechanisms underscore the dual risk posed by both low and high SUA levels on bone health, highlighting the need for a balanced consideration of physiological, pathological, and lifestyle factors.

The study holds the merit of being the first to ascertain the influence of SUA on bone health. The strength of our research lies in the expansive clinical database from which it draws and the sophisticated statistical analyses employed. Notably, the application of GSCF and threshold analysis using RCS has enriched our exploration and provided a deeper dimension to the research scope. However, while interpreting these findings, it is imperative to acknowledge certain limitations. Firstly, the inherent observational design limits our ability to establish causal relationships. Secondly, despite our rigorous attempts to adjust for a multitude of confounding factors, there remains the possibility of unmeasured confounders that could influence the observed associations. Additionally, the current study lacks data on lifestyle and dietary habits, which are known to significantly affect SUA metabolism and bone health, highlighting the need for their inclusion in future research. Moreover, we acknowledge that the accuracy of SUA measurements can be influenced by various factors, including temperature, pH, and reaction time. To minimize the impact of these variables, all samples were processed and analyzed under standardized conditions in our laboratory, following the manufacturer’s guidelines for the analyzer. Lastly, the study population was exclusively derived from a single center, which limits the generalizability of our results and urges caution in extrapolating these findings to broader populations.

## 5. Conclusions

In conclusion, this study found a close relationship between SUA levels and bone health in middle-aged and elderly hypertensive patients. Furthermore, it also highlights a significant U-shaped relationship between SUA levels and osteoporosis and future bone risk. Furthermore, the study offers valuable insights that could inform future therapeutic strategies for managing osteoporosis in this specific population. However, to validate these findings, further well-designed prospective studies are needed.

## Figures and Tables

**Table 1:** Characteristics of the study population based on serum uric acid tertiles.

Characteristic	T1	T2	T3	*p* Value
	<289 (µmol/L)	289–372 (µmol/L)	>372 (µmol/L)	
N	762	762	760	
Age (years)	58.36 ± 10.32	56.96 ± 11.01	54.02 ± 11.44	<0.001
Sex				<0.001
Female	544 (71.39%)	395 (51.84%)	230 (30.26%)	
Male	218 (28.61%)	368 (48.29%)	529 (69.61%)	
BMI (kg/m^2^)	26.35 ± 3.89	27.41 ± 3.74	27.48 ± 3.56	<0.001
SBP (mmHg)	144.67 ± 17.66	145.06 ± 17.09	145.14 ± 18.32	0.858
DBP (mmHg)	85.37 ± 12.48	86.38 ± 11.98	88.24 ± 13.54	<0.001
Current smoking (%)	96 (12.60%)	186 (24.41%)	288 (37.89%)	<0.001
Current drinking (%)	69 (9.06%)	133 (17.45%)	235 (30.92%)	<0.001
Menopausal (%)	401 (52.62%)	304 (39.90%)	181 (23.82%)	<0.001
**Medical history**				
PA	136 (17.85%)	90 (11.81%)	80 (10.53%)	<0.001
DM	244 (32.02%)	248 (32.55%)	204 (26.84%)	0.031
CHD	66 (8.66%)	53 (6.96%)	52 (6.84%)	0.319
Cancer	41 (5.38%)	41 (5.38%)	23 (3.03%)	0.028
**Laboratory tests**				
Serum potassium (mmol/L)	3.80 ± 0.35	3.90 ± 0.33	3.96 ± 0.33	<0.001
PTH (pg/mL)	50.30 (36.20–68.60)	53.30 (37.75–71.60)	50.00 (36.05–65.55)	<0.001
Serum calcium (mmol/L)	2.32 ± 0.55	2.35 ± 0.61	2.35 ± 0.54	0.564
25-hydroxyvitamin D (nmol/L)	19.31 (12.18–29.27)	18.32 (10.61–28.49)	19.11 (12.63–29.36)	0.161
Serum phosphorus (mmol/L)	1.15 ± 0.17	1.15 ± 0.17	1.18 ± 0.18	0.002
ALT (U/L)	23.00 (16.00–35.77)	20.50 (15.00–29.00)	24.00 (17.00–35.55)	<0.001
AST (U/L)	20.02 (16.60–26.00)	19.10 (16.00–24.78)	20.00 (16.70–25.77)	<0.001
TC (mmol/L)	4.53 ± 1.06	4.46 ± 1.09	4.58 ± 1.12	0.127
TG (mmol/L)	1.60 (1.15–2.39)	1.40 (1.00–2.16)	1.56 (1.15–2.38)	<0.001
Cr (µmol/L)	58.84 ± 15.37	63.94 ± 14.87	70.39 ± 16.25	<0.001
eGFR (mL/min/1.73 m^2^)	125.97 ± 63.61	121.50 ± 48.83	119.68 ± 57.98	0.087
ALP (U/L)	84.62 ± 29.81	82.28 ± 28.84	80.90 ± 28.84	0.042
TSH (uIU/mL)	2.17 (1.45–3.33)	2.31 (1.49–3.45)	2.09 (1.46–3.36)	0.008
FPG (mmol/L)	5.92 ± 2.06	5.88 ± 1.98	5.65 ± 1.83	0.014
SUA (µmol/L)	237.96 ± 35.98	328.75 ± 23.34	445.30 ± 64.89	<0.001
**Medications**				
Statins (%)	174 (22.83%)	145 (19.03%)	152 (20.00%)	0.16
Diuretics (%)	74 (9.71%)	81 (10.63%)	103 (13.55%)	0.045
Beta-blockers (%)	138 (18.11%)	140 (18.37%)	139 (18.29%)	0.992
Calcium channel blockers (%)	417 (54.72%)	413 (54.20%)	405 (53.29%)	0.866
ACEIs/ARBs (%)	298 (39.11%)	318 (41.73%)	356 (46.84%)	0.007
Oral hypoglycemic agents (%)	200 (26.25%)	185 (24.28%)	152 (20.00%)	0.014
Insulin (%)	60 (7.87%)	48 (6.30%)	42 (5.53%)	0.171
**DXA BMD T-scores**				
Lumbar 1-4	−1.20 ± 1.65	−0.51 ± 1.58	−0.44 ± 1.68	<0.001
Neck	−1.05 ± 1.10	−0.74 ± 1.00	−0.69 ± 1.01	<0.001
Total	−0.44 ± 1.12	−0.04 ± 1.01	−0.05 ± 1.03	<0.001
**FRAX score (%)**				
MOF	4.34 ± 3.12	3.31 ± 2.46	3.17 ± 2.37	<0.001
HF	1.81 ± 2.46	1.19 ± 1.93	1.21 ± 2.00	<0.001
**Osteoporosis (%)**	268 (35.17%)	115 (15.09%)	168 (22.11%)	<0.001

Data are presented as mean  ±  standard deviation, median (interquartile range), or as numbers and percentages. Abbreviations: BMI, body mass index; SBP, systolic blood pressure; DBP, diastolic blood pressure; PA, primary aldosteronism; DM, diabetes mellitus; CHD, coronary heart disease; PTH, parathyroid hormone; ALT, alanine transaminase; AST, aspartate transaminase; TC, total cholesterol; TG, triglyceride; Cr, creatinine; eGFR, estimation of glomerular filtration rate; ALP, alkaline phosphatase; TSH, thyroid stimulating hormone; FPG, fasting plasma glucose; SUA, serum uric acid; ACEIs, angiotensin-converting enzyme inhibitors; ARBs, angiotensin receptor blockers; BMD, bone mineral density; Neck, neck of the femur; Total, total femur; MOF, major osteoporotic fracture; HF, hip fracture.

**Table 2:** Relationship between serum uric acid levels and bone mineral density.

Exposure	Model 1	Model 2	Model 3	Model 4
	β (95% CI) *p* Value	β (95% CI) *p* Value	β (95% CI) *p* Value	β (95% CI) *p* Value
**Lumbar 1-4**				
SUA (per 10-µmol/L increase)	0.032 (0.025, 0.039) <0.001	0.017 (0.010, 0.024) 0.010	0.016 (0.008, 0.023) <0.001	0.010 (0.003, 0.018) 0.006
Tertiles of SUA				
Tertile 1	Reference	Reference	Reference	Reference
Tertile 2	0.687 (0.523, 0.852) <0.001	0.514 (0.351, 0.677) <0.001	0.504 (0.341, 0.667) <0.001	0.409 (0.248, 0.529) <0.001
Tertile 3	0.757 (0.592, 0.922) <0.001	0.435 (0.520, 0.959) <0.001	0.414 (0.242, 0.585) <0.001	0.271 (0.099, 0.443) 0.002
**Neck**				
SUA (per 10-µmol/L increase)	0.014 (0.009, 0.018) <0.001	0.008 (0.004, 0.013) <0.001	0.008 (0.004, 0.013) <0.001	0.004 (0.001, 0.008) 0.037
Tertiles of SUA				
Tertile 1	Reference	Reference	Reference	Reference
Tertile 2	0.309 (0.205, 0.413) <0.001	0.255 (0.155, 0.356) <0.001	0.253 (0.153, 0.354) <0.001	0.178 (0.080, 0.276) <0.001
Tertile 3	0.367 (0.262, 0.471) <0.001	0.258 (0.152, 0.364) <0.001	0.259 (0.153, 0.365) <0.001	0.139 (0.034, 0.244) 0.009
**Total**				
SUA (per 10-µmol/L increase)	0.013 (0.009, 0.018) <0.001	0.008 (0.003, 0.012) 0.002	0.008 (0.003, 0.013) 0.001	0.003 (−0.002, 0.007) 0.232
Tertiles of SUA				
Tertile 1	Reference	Reference	Reference	Reference
Tertile 2	0.396 (0.290, 0.501) <0.001	0.331 (0.228, 0.434) <0.001	0.330 (0.227, 0.433) <0.001	0.246 (0.147, 0.346) <0.001
Tertile 3	0.385 (0.279, 0.491) <0.001	0.283 (0.174, 0.392) <0.001	0.293 (0.185, 0.402) <0.001	0.158 (0.051, 0.264) 0.004

Model 1: age, sex, BMI, smoking status, and drinking status were adjusted. Model 2: Model 1 plus adjustment for DM, CHD, and cancer. Model 3: Model 2 plus adjustment for ALP, serum potassium, serum calcium, serum phosphorus, PTH, and 25-hydroxyvitamin D. Model 4: Model 3 plus adjustment for use of statins, diuretics, beta-blockers, calcium channel blockers, ACEIs/ARBs, oral hypoglycemic agents, and insulin. Abbreviations: SUA, serum uric acid; Neck, neck of the femur; Total, total femur; β, regression coefficient; CI, confidence interval. Other abbreviations, see Table 1.

**Table 3:** Relationship between serum uric acid levels and FRAX score.

Exposure	Model 1	Model 2	Model 3	Model 4
	β (95% CI) *p* Value	β (95% CI) *p* Value	β (95% CI) *p* Value	β (95% CI) *p* Value
**MOF**				
SUA (per 10-µmol/L increase)	−0.045 (−0.056, −0.033) <0.001	−0.026 (−0.037, −0.014) <0.001	−0.025 (−0.037, −0.014) <0.001	−0.013 (−0.024, −0.001) 0.031
Tertiles of SUA				
Tertile 1	Reference	Reference	Reference	Reference
Tertile 2	−1.033 (−1.301, −0.765) <0.001	−0.834 (−1.089, −0.579) <0.001	−0.826 (−1.081, −0.570) <0.001	−0.608 (−0.856, −0.361) <0.001
Tertile 3	−1.168 (−1.437, −0.900) <0.001	−0.758 (−1.027, −0.490) <0.001	−0.753 (−1.022, −0.484) <0.001	−0.413 (−0.678, −0.148) 0.002
**HF**				
SUA (per 10-µmol/L increase)	−0.020 (−0.030, −0.011) <0.001	−0.022 (−0.032, −0.012) <0.001	−0.022 (−0.032, −0.012) <0.001	−0.013 (−0.022, −0.003) 0.010
Tertiles of SUA				
Tertile 1	Reference	Reference	Reference	Reference
Tertile 2	−0.628 (−0.843, −0.412) <0.001	−0.632 (−0.844, 0.420) <0.001	−0.628 (−0.840, −0.416) <0.001	−0.467 (−674, −0.260) <0.001
Tertile 3	−0.600 (−0.815, −0.384) <0.001	−0.644 (−0.867, −0.421) <0.001	−0.645 (−0.868, −0.421) <0.001	−0.392 (−0614, −0.170) 0.001

Model 1: age, sex, BMI, smoking status, and drinking status were adjusted. Model 2: Model 1 plus adjustment for DM, CHD, and cancer. Model 3: Model 2 plus adjustment for ALP, serum potassium, serum calcium, serum phosphorus, PTH, and 25-hydroxyvitamin D. Model 4: Model 3 plus adjustment for use of statins, diuretics, beta-blockers, calcium channel blockers, ACEIs/ARBs, oral hypoglycemic agents, and insulin. Abbreviations: SUA, serum uric acid; MOF, major osteoporotic fracture; HF, hip fracture; β, regression coefficient; CI, confidence interval. Other abbreviations, see Table 1.

**Table 4:** Associations between serum uric acid levels and osteoporosis.

Exposure	Model 1	Model 2	Model 3	Model 4
	OR (95%CI) *p* Value	OR (95%CI) *p* Value	OR (95%CI) *p* Value	OR (95%CI) *p* Value
Tertiles of SUA				
Tertile 1	3.057 (2.386, 3.917) <0.001	2.705 (2.095, 3.493) <0.001	2.680 (2.074, 3.462) <0.001	2.427 (1.869, 3.153) <0.001
Tertile 2	Reference	Reference	Reference	Reference
Tertile 3	1.602 (1.232, 2.082) <0.001	1.911 (1.455, 2.510) <0.001	1.939 (1.475, 2.549) <0.001	2.146 (1.622, 2.841) <0.001

Model 1: age, sex, BMI, smoking status, and drinking status were adjusted. Model 2: Model 1 plus adjustment for DM, CHD, and cancer. Model 3: Model 2 plus adjustment for ALP, serum potassium, serum calcium, serum phosphorus, PTH, and 25-hydroxyvitamin D. Model 4: Model 3 plus adjustment for use of statins, diuretics, beta-blockers, calcium channel blockers, ACEIs/ARBs, oral hypoglycemic agents, and insulin. Abbreviations: SUA, serum uric acid; OR, odds ratio; CI, confidence interval. Other abbreviations, see Table 1.

## Data Availability

The data presented in this study are available on request from the corresponding author. The data are not publicly available due to privacy.

## Supplementary Materials

The following supporting information can be downloaded at https://www.mdpi.com/article/10.3390/metabo15010015/s1, Table S1: Covariance Diagnostics; Table S2: Comparison of characteristics between osteoporotic and non-osteoporotic groups; Table S3: Threshold effect analysis between serum uric acid levels and bone mineral density; Table S4: Threshold effect analysis between serum uric acid levels and FRAX score; Table S5: Analyzing the relationship between serum uric acid levels and osteoporosis using the RCS turning points; Table S6: Analyzing the relationship between serum uric acid and osteoporosis using the RCS turning points in females; Table S7: Analyzing the relationship between serum uric acid and osteoporosis using the RCS turning points in males; Table S8: Effect of each stratification factor on the relationship between serum uric acid levels and bone mineral density; Table S9: Effect of each stratification factor on the relationship between serum uric acid levels and osteoporosis; Table S10: Sensitivity analysis of the relationship between serum uric acid with bone mineral density, FRAX score, and osteoporosis was performed after excluding patients with cancer; Table S11: Sensitivity analysis of the relationship between serum uric acid with bone mineral density, FRAX score, and osteoporosis was performed after excluding patients with 25-hydroxyvitamin D < 20 nmol/L; Table S12: Sensitivity analysis of the relationship between serum uric acid with bone mineral density, FRAX score, and osteoporosis was performed after excluding patients with BMI > 30 kg/m^2^; Table S13: Sensitivity analysis of the relationship between serum uric acid with bone mineral density, FRAX score, and osteoporosis was performed excluding patients with taking diuretics; Table S14: Separate analyses of participants taking serum uric acid-lowering medications explore the relationship between serum uric acid and bone mineral density, FRAX Scores, and osteoporosis.
