# Correction: Song et al. Serum Uric Acid and Bone Health in Middle-Aged and Elderly Hypertensive Patients: A Potential U-Shaped Association and Implications for Future Fracture Risk. *Metabolites* 2025, *15*, 15

**DOI:** 10.3390/metabo16010089

**Published:** 2026-01-22

**Authors:** Shuaiwei Song, Xintian Cai, Junli Hu, Qing Zhu, Di Shen, Huimin Ma, Yingying Zhang, Rui Ma, Pan Zhou, Wenbo Yang, Jing Hong, Nanfang Li

**Affiliations:** 1 Hypertension Center, People’s Hospital of Xinjiang Uygur Autonomous Region, Urumqi 830001, China; 2 Xinjiang Hypertension Institute, Urumqi 830001, China; 3 NHC Key Laboratory of Hypertension Clinical Research, Urumqi 830001, China; 4 Key Laboratory of Xinjiang Uygur Autonomous Region “Hypertension Research Laboratory”, Urumqi 830001, China; 5 Xinjiang Clinical Medical Research Center for Hypertension (Cardio-Cerebrovascular) Diseases, Urumqi 830001, China

**References**

The authors would like to make the following correction to their published paper [1]. The change is as follows:

Reference [3] in the original publication is incorrectly cited and needs to be replaced with the following reference:

3. Yang, S.; Nguyen, N.D.; Center, J.R.; Eisman, J.A.; Nguyen, T.V. Association between hypertension and fragility fracture: A longitudinal study. *Osteoporos. Int.* **2014**, *25*, 97–103. https://doi.org/10.1007/s00198-013-2457-8.

The authors state that the scientific conclusions are unaffected. This correction was approved by the Academic Editor. The original publication has also been updated.
