# Supplementary material for: Serum Uric Acid and Bone Health in Middle-Aged and Elderly Hypertensive Patients: A Potential U-Shaped Association and Implications for Future Fracture Risk
Source: Metabolites. 2025 Jan 3;15(1):15. doi: 10.3390/metabo15010015 (PMC11766991; doi:10.3390/metabo15010015)
Supplement: Supplementary file 1 [file metabolites-15-00015-s001.zip › metabolites-3369922-supplementary.pdf]

metabolites

MDPI

Article

Serum Uric Acid and Bone Health in Middle-Aged and Elderly Hypertensive Patients: A Potential U-Shaped Association and Implications for Future Fracture Risk

Supplementary Material

1. Supplemental Material and Methods

Baseline Examination

Anthropometric measurements were taken by trained nurses. Data for height and weight were 3 acquired following a protocol standardized to an accuracy of 0.1 kg and 0.1 cm, respectively. Current smokers were defined as having smoked 100 cigarettes in their lifetime and currently smoking. Alcohol consumption was evaluated with questions regarding the types of alcoholic beverages, the frequency of alcohol consumption per week, and the usual amount consumed per occasion. Subjects who reported alcohol consumption >140 g/week for men and >70 g/week for women were deemed to have excessive alcohol consumption. Blood pressure was measured using a mercury sphygmomanometer after the patient had rested quietly for at least 10 minutes, and the average of multiple measurements was taken as the systolic and diastolic blood pressure values. All biochemical tests were measured by blood sampling after an overnight fast.

Collection of Serum Samples and Determination of Serum Uric Acid Levels

Participants had venous blood collected by trained nurses after an overnight fast. For serum samples, blood was drawn into tubes containing clot activators to facilitate clotting. After collection, the test tube was left at room temperature for 15–30 minutes to allow complete clot formation. Once clotting occurred, the test tube was centrifuged to separate the serum from the blood cells. The centrifuge spun the test tube at high speed, causing the heavier blood cells to settle at the bottom while the lighter serum remained at the top. The serum was then carefully separated from the clot and blood cells and transferred to a clean test tube for further analysis.

Serum uric acid levels were measured using an enzymatic method. A serum sample was mixed with a reagent containing uricase (Roche Cobas Uric Acid Reagent) and other chemicals that help detect hydrogen peroxide. Uricase breaks down uric acid, producing hydrogen peroxide. The hydrogen peroxide then reacts with a color-developing substrate, resulting in a color change. The intensity of this color change was measured spectrophotometrically. The absorbance was compared to a standard curve to determine the concentration of uric acid in the serum.

Definitions

Criteria for hypertension included self-reported hypertension, current use of antihypertensive medication, or systolic blood pressure (SBP) ≥ 140 mmHg and/or diastolic blood pressure (DBP) ≥ 90 mmHg recorded for at least three consecutive readings. diabetes was defined as fasting serum glucose ≥7.0 mmol/L, the 2-h serum glucose of the oral glucose tolerance test ≥11.1 mmol/L, or the current use of hypoglycaemic medication or insulin. coronary heart disease (CHD) was defined as a fatal or nonfatal myocardial infarction, unstable angina, and coronary revascularization. To identify primary aldosteronism (PA) according to the Endocrine Society's Clinical Practice Guidelines, patients were diagnosed with PA based on a PAC level of ≥ 12 ng/dL, an aldosterone-to-renin ratio ≥ 20, and a PAC value ≥ 10 ng/dL confirmed by saline infusion testing. Menopausal status was

defined according to World Health Organization recommendations, which regard amenorrhea lasting 12 months as indicative of the menopause. Cancer was based on self-report of physician diagnosis of cancer or malignancy and/or use of anticancer medications. The body mass index (BMI) was calculated as per the formula: Weight (kg)/Height^2 (m).

Bone Mineral Density Assessment

Dual-energy X-ray absorptiometry (DXA) scans were performed by two health technologists who were certified radiology technologists using the bone densitometer (Horizon Wi S/N302999M, Hologic, MA, USA). The DXA machine was calibrated daily with a phantom. Lumbar spine (L1–L4) and femoral regions (total femur; femur neck) scans of each subject were performed and analyzed upon test completion, with each scan taking approximately 5 min. The short-term in vivo coefficient of variation for the DXA machine was 1.8% for the lumbar spine and 1.2% for the femoral regions. The lumbar and femoral regions BMD were computed automatically by the DXA scanner.

Details of the statistical analyses.

Variables of baseline characteristics are shown as n (%) if categorical, mean (SD) if normally distributed, and median (interquartile range) if nonnormally distributed. To compare the characteristics among different SUA groups, the chi-square test was performed for categorical variables, and one-way analysis of variance, or the Kruskal-Wallis test, was performed for continuous variables with normal and skewed distributions.

Since there are some missing values in the sample data, we use multiple interpolation methods to fill in the missing values. The missing data in each dataset will be filled in by estimation model methods. Interpolation of the data is done using the corresponding R package.

Prior to any regression analysis, we tested multicollinearity among the predictors by checking for the variance inflation factor. Multicollinearity analyses revealed that variance inflation factors were smaller than 10 for all predictor variables, confirming that regression models were not affected by the presence of multicollinearity. Multivariable linear regression models were used to estimate the associations between SUA and BMD and FRAX scores, respectively. The association between SUA and osteoporosis was tested with multivariable logistic regression models. This study set four different models (Model 1: adjusted for age, sex, BMI, smoking status, and drinking status; Model 2: Model 1 plus adjustment for DM, CHD, and cancer. Model 3: Model 2 plus adjustment for ALP, serum potassium, serum calcium, serum phosphorus, PTH, and 25-hydroxyvitamin D. Model 4: Model 3 plus adjustment for use of statins, diuretics, beta-blockers, calcium channel blockers, ACEIs/ARBs, oral hypoglycemic agents, and insulin.). We use the restricted inverse square spline (RCS) (four nodes at the 5th, 35th, 65th, and 95th percentiles of the SUA distribution) and generalized smooth curve fitting was used to evaluate the nonlinear relationships. Moreover, RCS can better reflect the nonlinear dose-response relationship, and the inflection point can be further calculated by recursive algorithm. And, based on the inflection point, we can also carry out two stages of analysis before and after the inflection point. Finally, subgroup analysis was performed stratified by sex, age BMI, smoking status, and Menopausal.

We performed a series of sensitivity analyses to assess the robustness of our findings as follows: Recognizing the potential influence of malignancy on bone health among cancers, we first excluded all participants with cancer, yet our results remained robust. Additionally, considering the adverse effects of 25-hydroxyvitamin D deficiency on bone health, we excluded relevant patients, resulting in consistent findings. Additionally, considering the potential bone-related advantages of obesity, we removed participants with obesity from the analysis, which led to largely unchanged results. Lastly, understanding that some antihypertensive drugs might negatively affect SUA metabolism, we excluded participants who used diuretics, and this exclusion did not significantly alter the results. Further excluding participants taking beta-blockers, the findings remained robust.

All analyses were done using R (4.2.2). All P-values were two-sided, and P-values of <0.05 denoted statistical significance.

2. Supplementary Tables

Table S1. Covariance Diagnostics.

Variable	VIF
Sex	8.015
Age	1.832
BMI	1.161
SBP	1.632
DBP	1.999
Current smoking	1.758
Current drinking	1.505
Menopausal	6.569
Serum potassium	1.131
PTH	1.159
Serum calcium	1.029
25-hydroxyvitamin D	1.065
Serum phosphorus	1.116
ALT	3.893
AST	3.454
TC	1.247
TG	1.299
Cr	1.473
eGFR	1.057
FPG	1.638
ALP	1.184
TSH	1.065
DM	3.544
CHD	1.073
Statins	1.163
Diuretics	1.214
Beta-blockers	1.169
Calcium channel blockers	1.118
ACEIs/ARBs	1.262
Oral hypoglycemic agents	2.827
Insulin	1.206
PA	1.043
Cancer	1.023

VIF = $1/(1-R^2)$. VIF step-by-step screening method: Calculate the VIF of each variable. If the maximum VIF value is ≥ 10, remove the variable with the maximum VIF value. VIF: variance inflation factors. For other abbreviations, see Table 1.

Table S2. Comparison of characteristics between osteoporotic and non-osteoporotic groups.

**Characteristic**	**Non-osteoporotic**	**Osteoporotic**	P value
N	1733	551	
Age (years)	55.35±10.93	59.90±10.81	<0.001
Sex (%)			<0.001
Female	822 (47.43%)	347 (62.98%)	
Male	911 (52.57%)	204 (37.02%)	
BMI (kg/m^2)	27.37±3.74	26.16±3.71	<0.001
SBP (mmHg)	144.93±17.40	145.04±18.57	0.901
DBP (mmHg)	87.17±12.72	85.05±12.64	<0.001
Current smoking (%)	463 (26.72%)	107 (19.42%)	<0.001
Menopausal (%)	585 (33.76%)	301 (54.63%)	<0.001
**Medical history**			
PA (%)	256 (14.77%)	50 (9.07%)	<0.001
DM (%)	505 (29.14%)	191 (34.66%)	0.014
CHD (%)	119 (6.87%)	52 (9.44%)	0.046
Cancer (%)	79 (4.56%)	26 (4.72%)	0.883
**Laboratory tests**			
Serum potassium (mmol/L)	3.91±0.33	3.80±0.36	<0.001
PTH (pg/ml)	51.60±22.88	57.74±25.12	<0.001
Serum calcium (mmol/L)	2.35±0.58	2.32±0.54	0.426
25-hydroxyvitamin D (nmol/L)	21.43±11.73	20.52±12.14	0.117
Serum phosphorus (mmol/L)	1.16±0.17	1.14±0.18	0.019
ALT (U/L)	29.50±19.27	28.13±19.68	0.149
AST (U/L)	22.73±9.77	22.73±9.73	0.99
Cr (umol/L)	64.62±15.98	63.63±16.89	0.213
eGFR	123.65±58.06	118.40±54.12	0.06
ALP (U/L)	81.07±28.87	87.42±29.72	<0.001
TSH (uIU/mL)	2.55±1.54	2.60±1.60	0.536
FPG (mmol/L)	5.81±1.94	5.84±2.04	0.700
SUA (umol/L)	342.92±92.68	320.71±103.94	<0.001
**DXA BMD T-scores**			

Lumbar 1-4	−0.14±1.40	−2.53±1.05	<0.001
Neck	−0.54±0.94	−1.75±0.83	<0.001
Total	0.11±0.94	−1.10±0.94	<0.001
**FRAX scores (%)**			
MOF	2.64±1.26	6.65±3.68	<0.001
HF	0.71±0.83	3.60±3.30	<0.001
**Medications**			
Statins (%)	349 (20.14%)	122 (22.14%)	0.311
Diuretics (%)	193 (11.14%)	65 (11.80%)	0.67
Beta-blockers (%)	324 (18.70%)	93 (16.88%)	0.336
Calcium channel blockers (%)	929 (53.61%)	306 (55.54%)	0.429
ACEIs/ARBs (%)	733 (42.30%)	239 (43.38%)	0.655
Oral hypoglycemic agents (%)	390 (22.50%)	147 (26.68%)	0.044
Insulin (%)	101 (5.83%)	49 (8.89%)	0.011

Data are presented as mean ± standard deviation, or as numbers, and percentages. Abbreviations: see Table 1

Table S3. Threshold effect analysis between serum uric acid levels and bone mineral density.

Exposure	Lumbar 1-4	Neck	Total
SUA (per 10-μmol/L increase)	β (95% CI) P value	β (95% CI) P value	β (95% CI) P value
Turning point (μmol/L)	324	366	365
< =Turning point	0.069 (0.046, 0.091)<br><0.001	0.029 (0.019, 0.039)<br><0.001	0.037 (0.027, 0.047)<br><0.001
> Turning point	0.009 (−0.004, 0.022)<br>0.167	−0.005 (−0.015, 0.006)<br>0.408	−0.011 (−0.022, −0.001)<br>0.043
P for likelihood ratio test	<0.001	<0.001	<0.001

Age, sex, BMI, smoking status, drinking status, DM, CHD, cancer, ALP, serum potassium, serum calcium, serum phosphorus, PTH, 25-hydroxyvitamin D, statins, diuretics, beta-blockers, calcium channel blockers, ACEIs/ARBs, oral hypoglycemic agents, and insulin were adjusted. Abbreviations: SUA, serum uric acid; Neck, neck of the femur; Total, total femur; β, regression coefficient; CI, confidence interval. Other abbreviations, see Table 1.

Table S4. Threshold effect analysis between serum uric acid levels and FRAX score.

Exposure	MOF	HF
SUA (per 10-μmol/L increase)	β (95% CI) P value	β (95% CI) P value
Turning point (umol/L)	365	370
< =Turning point	−0.100 (−0.128, −0.073)<br><0.001	−0.060 (−0.081, −0.040)<br><0.001
> Turning point	0.016 (−0.008, 0.040)<br>0.199	0.026 (0.004, 0.047)<br>0.018
P for likelihood ratio test	<0.001	<0.001

Age, sex, BMI, smoking status, drinking status, DM, CHD, cancer, ALP, serum potassium, serum calcium, serum phosphorus, PTH, 25-hydroxyvitamin D, statins, diuretics, beta-blockers, calcium channel blockers, ACEIs/ARBs, oral hypoglycemic agents, and insulin were adjusted. Abbreviations: SUA, serum uric acid; MOF, major osteoporotic fracture; HF, hip fracture; β, regression coefficient; CI, confidence interval. Other abbreviations, see Table 1.

Table S5. Analyzing the relationship between serum uric acid levels and osteoporosis using the RCS turning points.

The Inflection Point of SUA	OR (95%CI) P value
Turning point	330 μmol/L
< 330 μmol/L (per 10-μmol/L increase)	0.903 (0.877–0.929) <0.001
>= 330 μmol/L (per 10-μmol/L increase)	1.035 (1.014–1.055) <0.001
P for likelihood ratio test	<0.001

Age, sex, BMI, smoking status, drinking status, DM, CHD, cancer, ALP, serum potassium, serum calcium, serum phosphorus, PTH, 25-hydroxyvitamin D, statins, diuretics, beta-blockers, calcium channel blockers, ACEIs/ARBs, oral hypoglycemic agents, and insulin were adjusted. Abbreviations: SUA, serum uric acid; RCS, restricted cubic splines; OR, odds ratio; CI, confidence interval. Other abbreviations, see Table 1.

Table S6. Analyzing the relationship between serum uric acid and osteoporosis using the RCS turning points in female.

The Inflection Point of SUA	OR (95%CI) P value
< =295 μmol/L (per 10-μmol/L increase)	0.927 (0.899–0.956) <0.001
> 295 μmol/L (per 10-μmol/L increase)	1.111 (1.062–1.163) <0.001
P for likelihood ratio test	<0.001

Age, BMI, smoking status, drinking status, DM, CHD, cancer, ALP, serum potassium, serum calcium, serum phosphorus, PTH, 25-hydroxyvitamin D, statins, diuretics, beta-blockers, calcium channel blockers, ACEIs/ARBs, oral hypoglycemic agents, and insulin were adjusted. Abbreviations: SUA, serum uric acid; RCS, restricted cubic splines; OR, odds ratio; CI, confidence interval. Other abbreviations, see Table 1.

Table S7. Analyzing the relationship between serum uric acid and osteoporosis using the RCS turning points in male.

The Inflection Point of SUA	OR (95%CI) P value
< =370 μmol/L (per 10-μmol/L increase)	0.874 (0.819–0.932) <0.001
> 370 μmol/L (per 10-μmol/L increase)	1.030 (1.004–1.055) 0.021
P for likelihood ratio test	<0.001

Age, BMI, smoking status, drinking status, DM, CHD, cancer, ALP, serum potassium, serum calcium, serum phosphorus, PTH, 25-hydroxyvitamin D, statins, diuretics, beta-blockers, calcium channel blockers, ACEIs/ARBs, oral hypoglycemic agents, and insulin were adjusted. Abbreviations: SUA, serum uric acid; RCS, restricted cubic splines; OR, odds ratio; CI, confidence interval. Other abbreviations, see Table 1.

Table S8. Effect of each stratification factor on the relationship between serum uric acid levels and bone mineral density.

BMD T-scores<br>SUA (per 10-μmol/L increase)	Lumbar 1-4<br>β (95%CI) P value	Neck<br>β (95%CI) P value	Total<br>β (95%CI) P value
**Sex**			
Female	0.012 (0.002, 0.023)<br>0.022	0.008 (0.001, 0.015)<br>0.025	0.012 (0.005, 0.019)<br>0.001
Male	0.026 (0.015, 0.036)<br>< 0.001	0.009 (0.004, 0.015)<br>0.001	0.005 (-0.001, 0.011)<br>0.090
**Age (years)**			
< 60	0.021 (0.012, 0.029)<br>< 0.001	0.003 (−0.002, 0.008)<br>0.248	0.002 (−0.003, 0.008)<br>0.396
>= 60	0.024 (0.011, 0.037)<br>< 0.001	0.008 (0.001, 0.016)<br>0.033	0.008 (0.000, 0.016)<br>0.042
**BMI (kg/m²)**			
<24	0.018 (0.003, 0.033)<br>0.016	0.005 (−0.004, 0.015)<br>0.276	0.002 (−0.007, 0.012)<br>0.628
>=24	0.021 (0.012, 0.029)<br>< 0.001	0.002 (−0.002, 0.007)<br>0.336	0.001 (−0.003, 0.006)<br>0.555
**Current smoking**			
No	0.017 (0.09, 0.025)<br>< 0.001	0.006 (0.001, 0.011)<br>0.028	0.007 (0.001, 0.012)<br>0.016
Yes	0.032 (0.018, 0.047)<br>< 0.001	0.007 (−0.001, 0.015)<br>0.089	0.001 (−0.007, 0.009)<br>0.719
**Menopausal**			
No	0.022 (0.001, 0.042)<br>0.037	0.014 (0.001, 0.027)<br>0.039	0.023 (0.009, 0.037)<br>0.001
Yes	0.010 (−0.002, 0.022)<br>0.096	0.007 (−0.001, 0.015)<br>0.107	0.009 (0.001, 0.017)<br>0.038

Age, sex, BMI, smoking status, drinking status, DM, CHD, cancer, ALP, serum potassium, serum calcium, serum phosphorus, PTH, 25-hydroxyvitamin D, statins, diuretics, beta-blockers, calcium channel blockers, ACEIs/ARBs, oral hypoglycemic agents, and insulin were adjusted. Abbreviations: SUA, serum uric acid; BMD, bone mineral density; Neck, neck of the femur; Total, total femur; β, regression coefficient; CI, confidence interval. Other abbreviations, see Table 1.

Table S9. Effect of each stratification factor on the relationship between serum uric acid levels and osteoporosis.

Osteoporosis<br>SUA (per 10-μmol/L increase)	OR (95%CI)	P value
Sex		
Female	0.984 (0.969, 0.999)	0.041
Male	0.989 (0.970, 1.002)	0.082
Age (years)		
<60	0.982 (0.969, 0.996)	0.012
>=60	0.972 (0.956, 0.989)	0.001
BMI (kg/m2)		
<24	0.984 (0.964, 1.005)	0.131
>=24	0.977 (0.965, 0.990)	< 0.001
Current smoking		
No	0.974 (0.962, 0.986)	< 0.001
Yes	0.991 (0.969, 1.013)	0.432
Menopausal		
No	0.950 (0.909, 0.993)	0.022
Yes	0.985 (0.968, 1.003)	0.095

Age, sex, BMI, smoking status, drinking status, DM, CHD, cancer, ALP, serum potassium, serum calcium, serum phosphorus, PTH, 25-hydroxyvitamin D, statins, diuretics, beta-blockers, calcium channel blockers, ACEIs/ARBs, oral hypoglycemic agents, and insulin were adjusted. Abbreviations: SUA, serum uric acid; OR, odds ratio; CI, confidence interval. Other abbreviations, see Table 1.

Table S10. Sensitivity analysis of the relationship between serum uric acid with bone mineral density, FRAX score, and osteoporosis was performed after excluding patients with cancer.

Exposure	Model 1<br>β/OR (95% CI) P value	Model 2<br>β/OR (95% CI) P value	Model 3<br>β/OR (95% CI) P value	Model 4<br>β/OR (95% CI) P value
**Lumbar 1-4**				
SUA (per 10-μmol/L increase)	0.034 (0.027, 0.041)<br><0.001	0.019 (0.011, 0.026)<br><0.001	0.014 (0.003, 0.024)<br>0.015	0.009 (−0.002, 0.020)<br>0.121
Tertiles of SUA				
Tertile 1	Reference	Reference	Reference	Reference
Tertile 2	0.687 (0.518, 0.855)<br><0.001	0.517 (0.351, 0.683)<br><0.001	0.505 (0.258, 0.753)<br><0.001	0.440 (0.194, 0.687)<br><0.001
Tertile 3	0.800 (0.633, 0.968)<br><0.001	0.473 (0.299, 0.647)<br><0.001	0.413 (0.159, 0.667)<br>0.001	0.297 (0.040, 0.554)<br>0.024
**Neck**				
SUA (per 10-μmol/L increase)	0.014 (0.010, 0.019)<br><0.001	0.009 (0.005, 0.014)<br><0.001	0.005 (0.002, 0.011)<br>0.034	0.002 (−0.006, 0.007)<br>0.825
Tertiles of SUA				
Tertile 1	Reference	Reference	Reference	Reference
Tertile 2	0.320 (0.214, 0.427)<br><0.001	0.266 (0.163, 0.369)<br><0.001	0.215 (0.068, 0.362)<br>0.004	0.170 (0.024, 0.315)<br>0.022
Tertile 3	0.383 (0.277, 0.489)<br><0.001	0.282 (0.174, 0.390)<br><0.001	0.221 (0.070, 0.372)<br>0.004	0.129 (−0.022, 0.281)<br>0.094
**Total**				
SUA (per 10-μmol/L increase)	0.014 (0.009, 0.018)<br><0.001	0.008 (0.003, 0.013)<br>0.001	0.004 (0.001, 0.003)<br>0.031	0.002 (−0.003, 0.005)<br>0.236
Tertiles of SUA				
Tertile 1	Reference	Reference	Reference	Reference
Tertile 2	0.410 (0.302, 0.519)<br><0.001	0.347 (0.242, 0.453)<br><0.001	0.290 (0.138, 0.442)<br><0.001	0.238 (0.087, 0.388)<br>0.002
Tertile 3	0.401 (0.293, 0.508)<br><0.001	0.299 (0.188, 0.409)<br><0.001	0.196 (0.039, 0.353)<br>0.014	0.104 (−0.064, 0.251)<br>0.139
**MOF**				

SUA (per 10-μmol/L increase)	−0.046 (−0.058, −0.034)<br><0.001	−0.024 (−0.036, −0.012)<br><0.001	−0.016 (−0.031, −0.007)<br>0.031	−0.004 (−0.019, 0.012)<br>0.613
Tertiles of SUA				
Tertile 1	Reference	Reference	Reference	Reference
Tertile 2	−1.038 (−1.312, −0.764)<br><0.001	−0.828 (−1.087, −0.568)<br><0.001	−0.847 (−1.201, −0.493)<br><0.001	−0.692 (−1.038, −0.346)<br><0.001
Tertile 3	−1.189 (−1.462, −0.916)<br><0.001	−0.742 (−1.013, −0.470)<br><0.001	−0.592 (−0.957, −0.228)<br>0.001	−0.302 (−0.662, 0.059)<br>0.101
**HF**				
SUA (per 10-μmol/L increase)	−0.021 (−0.030, −0.011)<br><0.001	−0.021 (−0.030, −0.011)<br>0.002	−0.017 (−0.028, −0.006)<br>0.014	−0.006 (−0.019, 0.006)<br>0.337
Tertiles of SUA				
Tertile 1	Reference	Reference	Reference	Reference
Tertile 2	−0.621 (−0.839, −403)<br><0.001	−0.618 (−0.831, −0.404)<br><0.001	−0.645 (−0.934, −0.357)<br><0.001	−0.532 (−0.816, −0.249)<br><0.001
Tertile 3	−0.606 (−0.823, −0.389)<br><0.001	−0.616 (−0.840, −0.393)<br><0.001	−0.500 (−0.797, −0.203)<br>0.001	−0.287 (−0.582, −0.009)<br>0.037
**Osteoporosis**				
Tertiles of SUA				
Tertile 1	3.222 (2.496, 4.160)<br><0.001	2.872 (2.207, 3.736)<br><0.001	2.882 (1.924, 4.315)<br><0.001	2.674 (1.771, 4.038)<br><0.001
Tertile 2	Reference	Reference	Reference	Reference
Tertile 3	1.594 (1.217, 2.087)<br><0.001	1.900 (1.436, 2.515)<br><0.001	2.414 (1.605, 3.631)<br><0.001	2.747 (1.803, 4.184)<br><0.001

Model 1: age, sex, BMI, smoking status, and drinking status were adjusted.

Model 2: Model 1 plus adjustment for DM, and CHD.

Model 3: Model 2 plus adjustment for ALP, serum potassium, serum calcium, serum phosphorus, PTH, and 25-hydroxyvitamin D.

Model 4: Model 3 plus adjustment for use of statins, diuretics, beta-blockers, calcium channel blockers, ACEIs/ARBs, oral hypoglycemic agents, and insulin.

Abbreviations: SUA, serum uric acid; MOF, major osteoporotic fracture; HF, hip fracture; β, regression coefficient; OR, odds ratio; CI, confidence interval. Other abbreviations, see Table 1.

Table S11. Sensitivity analysis of the relationship between serum uric acid with bone mineral density, FRAX score, and osteoporosis was performed after excluding patients with 25-hydroxyvitamin D < 20 nmol/L.

Exposure	Model 1<br>β/OR (95% CI) P value	Model 2<br>β/OR (95% CI) P value	Model 3<br>β/OR (95% CI) P value	Model 4<br>β/OR (95% CI) P value
**Lumbar 1-4**				
SUA (per 10-μmol/L increase)	0.028 (0.018, 0.038)<br><0.001	0.014 (0.003, 0.025)<br>0.010	0.017 (0.010, 0.025)<br><0.001	0.011 (0.004, 0.019)<br>0.003
Tertiles of SUA				
Tertile 1	Reference	Reference	Reference	Reference
Tertile 2	0.704 (0.460, 0.949)<br><0.001	0.507 (0.260, 0.755)<br><0.001	0.504 (0.339, 0.470)<br><0.001	0.399 (0.235, 0.563)<br><0.001
Tertile 3	0.700 (0.460, 0.941)<br><0.001	0.424 (0.169, 0.679)<br>0.001	0.445 (0.271, 0.619)<br><0.001	0.291 (0.116, 0.466)<br>0.001
**Neck**				
SUA (per 10-μmol/L increase)	0.008 (0.002, 0.014)<br>0.010	0.004 (−0.002, 0.011)<br>0.189	0.009 (0.004, 0.014)<br><0.001	0.004 (0.001, 0.009)<br>0.067
Tertiles of SUA				
Tertile 1	Reference	Reference	Reference	Reference
Tertile 2	0.280 (0.130, 0.430)<br><0.001	0.216 (0.069, 0.362)<br>0.004	0.265 (0.161, 0.368)<br><0.001	0.180 (0.079, 0.280)<br><0.001
Tertile 3	0.282 (0.134, 0.429)<br><0.001	0.221 (0.070, 0.371)<br><0.001	0.282 (0.173, 0.390)<br><0.001	0.153 (0.046, 0.260)<br>0.005
**Total**				
SUA (per 10−μmol/L increase)	0.007 (0.003, 0.012)<br>0.015	0.005 (0.003, 0.007)<br>0.040	0.009 (0.004, 0.013)<br>0.001	0.003 (−0.002, 0.008)<br>0.223
Tertiles of SUA				
Tertile 1	Reference	Reference	Reference	Reference
Tertile 2	0.360 (0.206, 0.513)<br><0.001	0.288 (0.135, 0.441)<br><0.001	0.349 (0.243, 0.454)<br><0.001	0.258 (0.156, 0.361)<br><0.001
Tertile 3	0.253 (0.101, 0.404)<br>0.001	0.192 (0.035, 0.349)<br>0.017	0.310 (0.199, 0.420)<br><0.001	0.169 (0.060, 0.278)<br>0.002
**MOF**				

SUA (per 10-μmol/L increase)	−0.038 (−0.053, −0.022)<br><0.001	−0.019 (−0.032, −0.008)<br>0.021	−0.024 (−0.036, −0.012)<br><0.001	−0.010 (−0.022, 0.002)<br>0.095
Tertiles of SUA				
Tertile 1	Reference	Reference	Reference	Reference
Tertile 2	−1.152 (−1.516, −0.787)<br><0.001	−0.851 (−1.205, −0.498)<br><0.001	−0.819 (−1.079, −0.559)<br><0.001	−0.581 (−0.833, −0.328)<br><0.001
Tertile 3	−1.041 (−1.400, −0.683)<br><0.001	−0.599 (−0.962, −0.235)<br>0.001	−0.736 (−1.008, −0.463)<br><0.001	−0.368 (−0.637, −0.099)<br>0.007
**HF**				
SUA (per 10-μmol/L increase)	−0.016 (−0.028, −0.007)<br>0.010	−0.015 (−0.028, −0.005)<br>0.012	−0.021 (−0.031, −0.011)<br>0.001	−0.010 (−0.020, −0.001)<br>0.041
Tertiles of SUA				
Tertile 1	Reference	Reference	Reference	Reference
Tertile 2	−0.684 (−0.973, −0.395)<br><0.001	−0.650 (−0.939, −0.362)<br><0.001	−0.614 (−0.828, −0.400)<br><0.001	0.246 (0.147, 0.346)<br><0.001
Tertile 3	−0.497 (−0.782, −0.213)<br>0.001	−0.502 (−0.798, −0.206)<br>0.001	−0.617 (−0.841, −0.393)<br><0.001	0.158 (0.051, 0.264)<br>0.004
**Osteoporosis**				
Tertiles of SUA				
Tertile 1	3.457 (2.344, 5.098)<br><0.001	2.885 (1.931, 4.310)<br><0.001	2.837 (2.179, 3.693)<br><0.001	2.533 (1.935, 3.316)<br><0.001
Tertile 2	Reference	Reference	Reference	Reference
Tertile 3	2.127 (1.433, 3.157)<br><0.001	2.387 (1.590, 3.583)<br><0.001	1.931 (1.458, 2.558)<br><0.001	2.130 (1.597, 2.841)<br>0.004

Model 1: age, sex, BMI, smoking status, and drinking status were adjusted.

Model 2: Model 1 plus adjustment for DM, CHD, and cancer.

Model 3: Model 2 plus adjustment for ALP, serum potassium, serum calcium, serum phosphorus, PTH, and 25-hydroxyvitamin D.

Model 4: Model 3 plus adjustment for use of statins, diuretics, beta-blockers, calcium channel blockers, ACEIs/ARBs, oral hypoglycemic agents, and insulin.

Abbreviations: SUA, serum uric acid; MOF, major osteoporotic fracture; HF, hip fracture; β, regression coefficient; OR, odds ratio; CI, confidence interval. Other abbreviations, see Table 1.

Table S12. Sensitivity analysis of the relationship between serum uric acid with bone mineral density, FRAX score, and osteoporosis was performed after excluding patients with BMI > 30 kg/m².

Exposure	Model 1<br>β/OR (95% CI) P value	Model 2<br>β/OR (95% CI) P value	Model 3<br>β/OR (95% CI) P value	Model 4<br>β/OR (95% CI) P value
**Lumbar 1-4**				
SUA (per 10-μmol/L increase)	0.031 (0.023, 0.039)<br><0.001	0.018 (0.010, 0.026)<br><0.001	0.017 (0.010, 0.025)<br><0.001	0.011 (0.003, 0.019)<br>0.009
Tertiles of SUA				
Tertile 1	Reference	Reference	Reference	Reference
Tertile 2	0.636 (0.456, 0.815)<br><0.001	0.485 (0.306, 0.663)<br><0.001	0.471 (0.293, 0.648)<br><0.001	0.395 (0.219, 0.570)<br><0.001
Tertile 3	0.724 (0.545, 0.903)<br><0.001	0.431 (0.244, 0.618)<br><0.001	0.422 (0.235, 0.608)<br><0.001	0.272 (0.085, 0.459)<br>0.004
**Neck**				
SUA (per 10-μmol/L increase)	0.014 (0.009, 0.019)<br><0.001	0.010 (0.005, 0.015)<br><0.001	0.010 (0.005, 0.015)<br><0.001	0.006 (0.001, 0.011)<br>0.020
Tertiles of SUA				
Tertile 1	Reference	Reference	Reference	Reference
Tertile 2	0.274 (0.161, 0.388)<br><0.001	0.260 (0.123, 0.397)<br><0.001	0.245 (0.135, 0.355)<br><0.001	0.188 (0.080, 0.296)<br>0.001
Tertile 3	0.371 (0.258, 0.484)<br><0.001	0.330 (0.115, 0.544)<br>0.003	0.304 (0.188, 0.420)<br><0.001	0.191 (0.076, 0.306)<br>0.001
**Total**				
SUA (per 10-μmol/L increase)	0.013 (0.008, 0.018)<br><0.001	0.009 (0.004, 0.014)<br>0.001	0.009 (0.004, 0.015)<br>0.001	0.004 (−0.001, 0.009)<br>0.100
Tertiles of SUA				
Tertile 1	Reference	Reference	Reference	Reference
Tertile 2	0.346 (0.230, 0.461)<br><0.001	0.353 (0.212, 0.494)<br><0.001	0.310 (0.197, 0.423)<br><0.001	0.250 (0.141, 0.360)<br><0.001
Tertile 3	0.370 (0.255, 0.485)<br><0.001	0.395 (0.174, 0.616)<br>0.017	0.306 (0.186, 0.425)<br><0.001	0.179 (0.062, 0.297)<br>0.003
**MOF**				
SUA (per 10-μmol/L increase)	−0.038 (−0.053, −0.022)	−0.031 (−0.045, −0.017)	−0.031 (−0.045, −0.017)	−0.017 (−0.031, 0.003)

	<0.001	<0.001	<0.001	0.016
Tertiles of SUA				
Tertile 1	Reference	Reference	Reference	Reference
Tertile 2	−0.995 (−1.312, −0.678)<br><0.001	−0.860 (−1.166, −0.553)<br><0.001	−0.856 (−1.162, −0.549)<br><0.001	−0.664 (−0.961, −0.367)<br><0.001
Tertile 3	−1.201 (−1.517, −0.814)<br><0.001	−0.869 (−1.190, −0.548)<br><0.001	−0.880 (−1.202, −0.580)<br><0.001	−0.511 (−0.829, −0.194)<br>0.002
**HF**				
SUA (per 10-μmol/L increase)	−0.022 (−0.033, −0.010)<br><0.001	−0.026 (−0.038, −0.014)<br><0.001	−0.027 (−0.038, −0.015)<br><0.001	−0.016 (−0.027, −0.004)<br>0.008
Tertiles of SUA				
Tertile 1	Reference	Reference	Reference	Reference
Tertile 2	−0.623 (−0.882, −0.364)<br><0.001	−0.678 (−0.934, −0.422)<br><0.001	−0.679 (−0.936, −0.423)<br><0.001	−0.536 (−0.787, −0.285)<br><0.001
Tertile 3	−0.632 (−0.891, −0.374)<br>0.001	−0.741 (−1.009, −0.473)<br><0.001	−0.750 (−1.109, −0.481)<br><0.001	−0.473 (−0.740, −0.205)<br>0.001
**Osteoporosis**				
Tertiles of SUA				
Tertile 1	2.794 (2.136, 3.653)<br><0.001	2.510 (1.905, 3.308)<br><0.001	2.468 (1.872, 3.256)<br><0.001	2.263 (1.706, 3.000)<br><0.001
Tertile 2	Reference	Reference	Reference	Reference
Tertile 3	1.465 (1.100, 1.952)<br><0.001	1.720 (1.279, 2.313)<br><0.001	1.734 (1.288, 2.334)<br><0.001	1.930 (1.424, 2.617)<br><0.001

Model 1: age, sex, BMI, smoking status, and drinking status were adjusted.

Model 2: Model 1 plus adjustment for DM, CHD, and cancer.

Model 3: Model 2 plus adjustment for ALP, serum potassium, serum calcium, serum phosphorus, PTH, and 25−hydroxyvitamin D.

Model 4: Model 3 plus adjustment for use statins, diuretics, beta-blockers, calcium channel blockers, ACEIs/ARBs, oral hypoglycemic agents, and insulin.

Abbreviations: SUA, serum uric acid; MOF, major osteoporotic fracture; HF, hip fracture; β, regression coefficient; OR, odds ratio; CI, confidence interval. Other abbreviations, see Table 1.

Table S13. Sensitivity analysis of the relationship between serum uric acid with bone mineral density, FRAX score, and osteoporosis was performed excluding patients with taking diuretics.

Exposure	Model 1 β/OR (95% CI) P value	Model 2 β/OR (95% CI) P value	Model 3 β/OR (95% CI) P value	Model 4 β/OR (95% CI) P value
**Lumbar 1-4**				
SUA (per 10-μmol/L increase)	0.031 (0.023, 0.039) <0.001	0.018 (0.010, 0.026) <0.001	0.017 (0.010, 0.025) <0.001	0.011 (0.007, 0.019) <0.001
Tertiles of SUA				
Tertile 1	Reference	Reference	Reference	Reference
Tertile 2	0.636 (0.456, 0.815) <0.001	0.485 (0.306, 0.663) <0.001	0.471 (0.293, 0.648) <0.001	0.395 (0.219, 0.570) <0.001
Tertile 3	0.724 (0.545, 0.903) <0.001	0.431 (0.244, 0.618) <0.001	0.422 (0.235, 0.608) <0.001	0.272 (0.085, 0.459) <0.001
**Neck**				
SUA (per 10-μmol/L increase)	0.014 (0.009, 0.019) <0.001	0.014 (0.009, 0.018) <0.001	0.010 (0.005, 0.014) <0.001	0.009 (0.005, 0.014) <0.001
Tertiles of SUA				
Tertile 1	Reference	Reference	Reference	Reference
Tertile 2	0.289 (0.179, 0.399) <0.001	0.288 (0.178, 0.396) <0.001	0.233 (0.125, 0.342) <0.001	0.229 (0.120, 0.337) <0.001
Tertile 3	0.371 (0.259, 0.482) <0.001	0.367 (0.256, 0.479) <0.001	0.263 (0.152, 0.374) <0.001	0.260 (0.149, 0.371) <0.001
**Total**				
SUA (per 10-μmol/L increase)	0.013 (0.008, 0.018) <0.001	0.014 (0.009, 0.018) <0.001	0.008 (0.004, 0.014) <0.001	0.008 (0.003, 0.013) 0.005
Tertiles of SUA				
Tertile 1	Reference	Reference	Reference	Reference
Tertile 2	0.381 (0.269, 0.493) <0.001	0.378 (0.265, 0.489) <0.001	0.377 (0.265, 0.489) <0.001	0.304 (0.195, 0.413) <0.001
Tertile 3	0.388 (0.274, 0.501) <0.001	0.391 (0.278, 0.505) <0.001	0.391 (0.278, 0.505) <0.001	0.253 (0.140, 0.365) <0.001
**MOF**				
SUA (per 10-μmol/L increase)	−0.045 (−0.057, −0.033)	−0.044 (−0.056, −0.032)	−0.032 (−0.044, −0.020)	−0.031 (−0.043, −0.019)

	<0.001	<0.001	<0.001	<0.001
Tertiles of SUA				
Tertile 1	Reference	Reference	Reference	Reference
Tertile 2	−0.957 (−1.239, −0.674)<br><0.001	−0.949 (−1.231, −0.666)<br><0.001	−0.779 (−1.057, −0.501)<br><0.001	−0.769 (−1.047, −0.491)<br><0.001
Tertile 3	−1.151 (−1.437, −0.865)<br><0.001	−1.127 (−1.413, −0.841)<br><0.001	−0.823 (−1.108, −0.538)<br><0.001	−0.800 (−1.085, −0.515)<br><0.001
**HF**				
SUA (per 10-μmol/L increase)	−0.021 (−0.031, −0.011)<br><0.001	−0.020 (−0.030, −0.011)<br><0.001	−0.017 (−0.027, −0.010)<br><0.001	−0.014 (−0.025, −0.003)<br>0.007
Tertiles of SUA				
Tertile 1	Reference	Reference	Reference	Reference
Tertile 2	−0.585 (−0.813, −0.356)<br><0.001	−0.581 (−0.810, −0.352)<br><0.001	−0.451 (−0.677, −0.226)<br><0.001	−0.448 (−0.674, −0.222)<br><0.001
Tertile 3	−0.596 (−0.827, −0.365)<br>0.001	−0.587 (−0.819, −0.356)<br><0.001	−0.352 (−0.583, −0.321)<br><0.001	−0.342 (−0.574, −0.210)<br><0.001
**Osteoporosis**				
Tertiles of SUA				
Tertile 1	2.491 (1.903, 3.264)<br><0.001	2.463 (1.882, 3.234)<br><0.001	2.209 (1.678, 2.923)<br><0.001	2.224 (1.688, 2.927)<br><0.001
Tertile 2	Reference	Reference	Reference	Reference
Tertile 3	1.847 (1.392, 2.471)<br><0.001	1.881 (1.412, 2.516)<br><0.001	2.094 (1.558, 2.816)<br><0.001	2.087 (1.563, 2.816)<br><0.001

Model 1: age, sex, BMI, smoking status, and drinking status were adjusted.

Model 2: Model 1 plus adjustment for DM, CHD, and cancer.

Model 3: Model 2 plus adjustment for ALP, serum potassium, serum calcium, serum phosphorus, PTH, and 25-hydroxyvitamin D.

Model 4: Model 3 plus adjustment for use statins, beta-blockers, calcium channel blockers, ACEIs/ARBs, oral hypoglycemic agents, and insulin.

Abbreviations: SUA, serum uric acid; MOF, major osteoporotic fracture; HF, hip fracture; β, regression coefficient; OR, odds ratio; CI, confidence interval. Other abbreviations, see Table 1.

Table S14. Separate analyses of participants taking serum uric acid-lowering medications explore the relationship between serum uric acid and bone mineral density, FRAX Scores, and osteoporosis.

Exposure	Model 1<br>β/OR (95% CI) P value	Model 2<br>β/OR (95% CI) P value	Model 3<br>β/OR (95% CI) P value	Model 4<br>β/OR (95% CI) P value
**Lumbar 1-4**				
SUA (per 10-μmol/L increase)	0.030 (0.022, 0.038)<br><0.001	0.022 (0.015, 0.030)<br><0.001	0.018 (0.011, 0.026)<br><0.001	0.018 (0.010, 0.025)<br><0.001
Tertiles of SUA				
Tertile 1	Reference	Reference	Reference	Reference
Tertile 2	0.623 (0.441, 0.804)<br><0.001	0.548 (0.374, 0.723)<br><0.001	0.479 (0.305, 0.652)<br><0.001	0.470 (0.296, 0.645)<br><0.001
Tertile 3	0.738 (0.556, 0.919)<br><0.001	0.568 (0.393, 0.744)<br><0.001	0.459 (0.282, 0.635)<br><0.001	0.453 (0.275, 0.630)<br><0.001
**Neck**				
SUA (per 10-μmol/L increase)	0.014 (0.009, 0.019)<br><0.001	0.014 (0.009, 0.019)<br><0.001	0.010 (0.006, 0.015)<br><0.001	0.010 (0.006, 0.015)<br><0.001
Tertiles of SUA				
Tertile 1	Reference	Reference	Reference	Reference
Tertile 2	0.280 (0.165, 0.394)<br><0.001	0.278 (0.163, 0.392)<br><0.001	0.220 (0.108, 0.333)<br><0.001	0.210 (0.097, 0.323)<br><0.001
Tertile 3	0.383 (0.268, 0.498)<br><0.001	0.381 (0.266, 0.496)<br><0.001	0.289 (0.175, 0.404)<br><0.001	0.287 (0.172, 0.401)<br><0.001
**Total**				
SUA (per 10-μmol/L increase)	0.012 (0.008, 0.018)<br><0.001	0.013 (0.008, 0.018)<br><0.001	0.008 (0.005, 0.015)<br><0.001	0.008 (0.003, 0.013)<br><0.001
Tertiles of SUA				
Tertile 1	Reference	Reference	Reference	Reference
Tertile 2	0.354 (0.235, 0.473)<br><0.001	0.350 (0.232, 0.496)<br><0.001	0.270 (0.155, 0.386)<br><0.001	0.263 (0.147, 0.378)<br><0.001
Tertile 3	0.381 (0.263, 0.500)<br><0.001	0.385 (0.266, 0.504)<br><0.001	0.257 (0.141, 0.374)<br><0.001	0.255 (0.138, 0.373)<br><0.001
**MOF**				
SUA (per 10-μmol/L increase)	−0.045 (−0.058, −0.032)	−0.044 (−0.058, −0.031)	−0.034 (−0.047, −0.020)	−0.033 (−0.046, −0.019)

	<0.001	<0.001	<0.001	<0.001
Tertiles of SUA				
Tertile 1	Reference	Reference	Reference	Reference
Tertile 2	−1.035 (−1.341, −0.729)<br><0.001	−1.021 (−1.327, −0.714)<br><0.001	−0.839 (−1.141, −0.538)<br><0.001	−0.811 (−1.113, −0.509)<br><0.001
Tertile 3	−1.197 (−1.504, −0.891)<br><0.001	−1.181 (−1.488, −0.874)<br><0.001	−0.896 (−1.202, −0.590)<br><0.001	−0.872 (−1.178, −0.565)<br><0.001
**HF**				
SUA (per 10-μmol/L increase)	−0.020 (−0.031, −0.009)<br><0.001	−0.020 (−0.031, −0.009)<br><0.001	−0.012 (−0.022, −0.007)<br><0.001	−0.012 (−0.022, −0.005)<br>0.005
Tertiles of SUA				
Tertile 1	Reference	Reference	Reference	Reference
Tertile 2	−0.650 (−0.896, −0.404)<br><0.001	−0.644 (−0.891, −0.398)<br><0.001	−0.608 (−0.891, −0.385)<br><0.001	−0.594 (−0.768, −0.351)<br><0.001
Tertile 3	−0.614 (−0.860, −0.368)<br><0.001	−0.609 (−0.856, −0.362)<br><0.001	−0.592 (−0.739, −0.354)<br><0.001	−0.581 (−0.649, −0.303)<br><0.001
**Osteoporosis**				
Tertiles of SUA				
Tertile 1	2.327 (1.773, 3.076)<br><0.001	2.312 (1.764, 3.064)<br><0.001	2.104 (1.578, 2.794)<br><0.001	2.103 (1.578, 2.794)<br><0.001
Tertile 2	Reference	Reference	Reference	Reference
Tertile 3	1.718 (1.275, 2.321)<br><0.001	1.758 (1.313, 2.374)<br><0.001	1.923 (1.417, 2.612)<br><0.001	1.891 (1.401, 2.568)<br><0.001

Model 1: age, sex, BMI, smoking status, and drinking status were adjusted.

Model 2: Model 1 plus adjustment for DM, CHD, and cancer.

Model 3: Model 2 plus adjustment for ALP, serum potassium, serum calcium, serum phosphorus, PTH, and 25-hydroxyvitamin D.

Model 4: Model 3 plus adjustment for use statins, diuretics, calcium channel blockers, ACEIs/ARBs, oral hypoglycemic agents, and insulin.

Abbreviations: SUA, serum uric acid; MOF, major osteoporotic fracture; HF, hip fracture; β, regression coefficient; OR, odds ratio; CI, confidence interval. Other abbreviations, see Table 1.
